# A natural experiment to examine the impact of park renewal on park-use and park-based physical activity in a disadvantaged neighbourhood: the REVAMP study methods

**DOI:** 10.1186/1471-2458-14-600

**Published:** 2014-06-13

**Authors:** Jenny Veitch, Jo Salmon, Alison Carver, Anna Timperio, David Crawford, Elly Fletcher, Billie Giles-Corti

**Affiliations:** 1 Centre for Physical Activity and Nutrition Research, Deakin University, 221 Burwood Highway, Burwood, VIC 3125, Australia; 2 Melbourne School of Population and Global Health, The University of Melbourne, Melbourne, Australia

**Keywords:** Natural experiment, Park refurbishment, Physical activity, Park use

## Abstract

**Background:**

Modifying the built environment by improving parks is potentially a sustainable way to increase population level physical activity. Despite considerable investment in parks and park renovations, few natural experiments on the impact of improving amenities on park use and park-based physical activity have been conducted. REVAMP is a natural experiment that aims to examine whether park improvement increases overall park usage, park-based physical activity and active travel to and from the park in the intervention compared with the control park over a two-year period; and to identify which specific aspects of the park refurbishment attracts park visitors and encourages park users to be more active. This paper describes the methods of the REVAMP study.

**Methods:**

The intervention park is a large regional park (329 hectares) located in a low socio-economic status (SES) area in the north-eastern suburbs of Melbourne, Australia. The control park is a regional park (120 hectares) located in a high SES area in the eastern suburbs of Melbourne. Multiple methodologies to evaluate the impact of the park renovation are being employed including: cross-sectional surveys of local residents living near the two parks, direct observations of park users, intercept surveys with park users, and electronic monitoring of path usage and car traffic within the parks. Baseline measures were conducted in April-May 2013 (T1), and an innovative play space suitable for children of all ages and abilities was installed at the intervention park between September 2013 and February 2014. Follow-up measures will be repeated in April-May 2014 (T2) and April-May 2015 (T3). All methodologies will be completed at both the intervention and control parks at all three time-points, with the exception of the cross-sectional survey of local residents which will only be conducted at T1 and T3.

**Conclusion:**

This research will inform future park developments, and will contribute to creating an evidence base of the impact of park refurbishment, and the development of natural experiment methodology.

**Trial Registration:**

Current controlled trial ISRCTN50745547, registration date 11.1.2014.

## Background

Modifying parks by improving access and optimizing their design to encourage activity is potentially a sustainable way to increase population-level physical activity. Parks are located in most neighbourhoods, they are generally free to access, offer a variety of opportunities for physical activity and can serve diverse populations [1]. Research shows that park availability, proximity and access are associated with higher overall levels of physical activity [2] and spending time in parks can be restorative and beneficial to mental health [3]. There is also evidence that physical activity undertaken in parks or green-spaces may have greater psychological and physiological benefits than physical activity in other settings [4,5]. Given that physical inactivity is a major contributor to the burden of chronic disease, including cardiovascular disease, diabetes, and overweight and obesity [6,7], understanding how to attract residents to parks and encourage park users to be physically active is an important public health goal.

Parks may encourage physical activity in two ways: as an important destination to which people walk or cycle (i.e. active transport) [8] and as a destination for physical activity whereby the presence of a high quality park may encourage increased physical activity within the park [9]. Both of these ‘opportunities’ for physical activity may make substantial contributions to overall physical activity levels and therefore benefit public health. Just having a park located close to home; however, may be necessary but insufficient for promoting physical activity. Observational studies of park use in the USA have shown that more than half of park users engaged in sedentary behaviour (primarily sitting) during their park visit [10,11]. A number of studies have found park quality and specific park features to be a major factor associated with achieving recommended levels of activity [9,12-14]. However, an Australian study found that a wider range of activities, including sedentary behaviours such as picnics and sitting, took place in more attractive parks [12]. While this may benefit mental health, without active transportation to the park, it will have little impact on physical activity and thus risk of chronic disease. Hence, simply making parks attractive for users may not be enough to increase community-wide physical activity levels. Understanding specific park features that attract users and encourage park-based physical activity is therefore important.

Improving parks may be particularly advantageous for increasing physical activity levels among disadvantaged populations where residents do less recreational physical activity [15] and are at an increased risk of inactivity and associated poor health outcomes [16]. For example, in the UK the increased risk of all-cause and circulatory disease mortality related to socio-economic disadvantage has been shown to be lower in those living in the ‘greenest’ areas (i.e., areas with more parks) compared with those who have less exposure to parks [17]. Although previous research in Melbourne Australia, showed no differences in park access according to area-level socioeconomic disadvantage (adjusted for population density) [18], parks in low socio-economic status (SES) areas have fewer amenities and features likely to promote physical activity among children than do parks in higher SES areas [19]. The importance of conducting natural experiments on open spaces for disadvantaged populations has recently been recognised [20].

Natural experiments enable researchers, in the absence of randomised controlled trials, to evaluate the effectiveness of ‘real world’ policy interventions that have not been manipulated by the researcher [21]. Natural experiments have been identified as a priority for investigating causal associations between the built environment and physical activity [22]; both in terms of increasing the evidence-base regarding environmental determinants of physical activity, and for identifying effective environmental interventions and policies to promote physical activity [23]. Due to the substantial financial costs and logistical challenges of conducting research involving major modification of the built environment, there is little research in this area. Rather, these studies need to be opportunistic. A few natural experiments examining physical activity have monitored the development of neighbourhood greenway/trails [24], sporting playfields [25], walking/cycling trails [26,27], and the implementation of new residential development codes [28], with results showing some increases in overall path use [24] and visits to the sporting playfields [25]. However, limited research has focused specifically on parks.

In the USA, a study examining the impact of improvements to five parks showed a decline in park use in both the intervention and control parks although this may have been attributable to simultaneous cuts to within-park programming [29]. Another study in USA that evaluated the impact of installing outdoor exercise equipment in 12 parks showed a non-significant increase in park usage in the intervention compared with control parks [30]. However, the follow-up observations were conducted at different times of the year and the period between measurements were not consistent between parks. In Australia, a study examining impact of playground renovation on usage and physical activity found no detectable difference in use or in children engaged in moderate- to vigorous-intensity physical activity at follow-up [31]. In contrast, another natural experiment study in Australia, which examined the impact of improvements at a small neighbourhood park on park usage and park-based physical activity, showed significant increases in park use (T1 = 235, T3 = 985) and people engaged in park-based vigorous activity (T1 = 38, T3 = 257) following park refurbishment [32]. That study provided evidence that park improvement has the potential to dramatically increase park usage; however, it focused only on improvements involving the installation of a small playground, walking track, and off-leash dog walking area in one park. Further evidence of the impact of park refurbishment in more diverse parks, in terms of size and features, and in neighbourhoods with residents of varying socio-demographic profiles is needed.

In summary, if neighbourhood parks are to attract more users and facilitate increased physical activity, research is needed to better understand park use, the activities park users engage in whilst at the park, and what improvements to park facilities result in increased park use and physical activity levels. Despite the considerable public funds invested in park renovations, little research evidence exists examining whether improving amenities in parks actually increases park use or park-based physical activity. This paper presents an overview of the aims, methodology and design of the REVAMP study, which seeks to address this gap.

### The REVAMP study

#### The intervention

REVAMP (Recording and EValuating Activity in a Modified Park) is a natural experiment examining the impact of the refurbishment of a large regional park (Brimbank Park) located in an area of low SES of Melbourne, Victoria, Australia. Specifically, the three main research objectives are to:

1. Examine whether park improvement increases overall park usage in the intervention park compared with the control park;

2. Examine whether park improvement increases the proportion of local residents engaging in park-based physical activity and active travel to and from the park in the intervention compared with the control park; and

3. Identify the specific aspects of the park refurbishment that attract visitors to the park and encourage park users to be more active.

The refurbishment of Brimbank Park commenced in September 2013 and was completed in February 2014. It involved the installation of an innovative play space suitable for children of all ages and abilities. This circumstance provided a rare opportunity to undertake a natural experiment, to observe changes that occur in a population before and after the park environment has been altered. Whilst the redevelopment will focus on the installation of a play space for children, it is important to assess the broader impact of the refurbishment on parents, families, adults and older adults.

As well as being accessible by road, Brimbank Park (329 hectares) is accessible via a shared path for walking and cycling that stretches 28 km north-west from Melbourne’s central business district (CBD). Within the park is a further 4.3 km shared path circuit. The City of Brimbank has a total population of almost 190,000 residents. The demographic profile of residents is diverse, with a high proportion of children aged 0–9 years (12.7%), a growing indigenous population (currently 0.4%), and a high proportion of residents born overseas (49.6%) [33].

#### The control park

Westerfolds Park (120 hectares) is located 22 km east of Melbourne’s CBD. This park was selected to match as closely as possible to the intervention park prior to refurbishment using the following criteria: 1) park features (i.e. mainly open spaces and picnic areas); 2) park size; 3) accessibility via a shared walking/cycling path; and 4) availability of a sealed walking/cycling path within the park. The control park is located in a more socio-economically advantaged area of Melbourne; however, it was not possible to find a matching large park in a disadvantaged area with similar features to the intervention park. Importantly, there are no planned improvements or changes to the control park during the study period.

#### The funding partners

This project is funded by the Australian Research Council and includes four partners: Parks Victoria, the Victorian Health Promotion Foundation (VicHealth), Brimbank City Council and City West Water. Parks Victoria funded and was responsible for the refurbishment of Brimbank Park. The inclusion of the partners in this project is important as they will play a critical role in disseminating and ensuring uptake of the results at the local, state and national levels. VicHealth and Parks Victoria play significant roles in preventative health in Victoria through the provision of health initiatives and also act as agents of change through their influence on government policy as well as practice. The inclusion of Brimbank City Council and City West Water is also significant due to their considerable local investment in public open space development and their responsibilities for meeting resident needs. Ethics approval for this study was granted by the Deakin University Human Ethics Advisory Group, the Department of Education and Early Childhood Development and the Catholic Education Office Melbourne.

## Methods

Baseline data collection occurred in April-May 2013 (T1, prior to the refurbishment commencing), in both the intervention and control parks. Follow-up will be conducted at both parks at one-year intervals, Time 2 (T2, 2014) and Time 3 (T3, 2015) with each data collection to take place at the same time of the year to account for seasonal effects. The inclusion of two measures post-refurbishment allows time for residents to become aware of the new facilities [32]. To address the study aims, multiple methodologies will be employed including: cross-sectional surveys of local residents living nearby the two parks, direct observations of park users, intercept surveys with park users, and electronic monitoring of path usage within the parks and of vehicles entering and exiting on-site car parks.

### Neighbourhood survey

Cross-sectional surveys of adults living within 5 km of the two parks will be employed at T1 and T3 to determine population changes in park use. Surveys will not be distributed at T2 as there is insufficient time between completion of the park refurbishment and T2 data collection to obtain data on usual park visitation. A longitudinal design is typically used for measuring within-person increases in behaviour; however, a repeated cross-sectional survey was considered the most appropriate for determining population-level increases. As the intervention is a play-space it is important to recruit families with children aged between 2–15 years. To obtain participants with these demographic characteristics, recruitment was via two methods: 1) families with children attending pre-schools, primary and secondary schools located within 3 km of each park; and 2) a postal survey from the local City Council to households located within 5 km of each park.

#### Recruitment via schools

For baseline measures, pre-schools, primary (elementary) and secondary government and Catholic schools located within a 3 km buffer surrounding the two parks were emailed and contacted by telephone and invited to participate. Recruitment of schools continued until six pre-schools, ten primary schools and two secondary schools were recruited. This number of schools resulted in approximately 5,000 eligible families (2,500 from each area) with children aged 2–15 years attending schools in both the Brimbank and Westerfolds areas. Once the schools consented to participate it was arranged for a survey to be sent home to: all families at each preschool with a child aged 2 years or older; every family at each primary school; and all families at each secondary school with an adolescent in school years 7–9. The survey envelope included a plain language statement, a consent form, a survey, and a reply-paid envelope for survey return.

Information on the study was placed in school newsletters in the week prior to surveys being distributed. In order to encourage survey return, reminder postcards were sent home to each family two weeks and six weeks after the surveys had been distributed and where possible a further reminder was placed in school newsletters.

#### Recruitment via postal survey

A random selection of 5000 residents (n = 2500 from each park area) who lived within a 5 km buffer of the two parks were identified from the two City Councils within which the parks were located. Pre-notification postcards were sent from the Council mail room to all residents one week prior to the survey being posted. A survey addressed ‘to the resident’ was then posted to each resident in a Council envelope with a letter from the Council inviting their participation in the study, a plain language statement, a consent form and a reply-paid envelope for survey return. In order to encourage survey return, follow-up reminder postcards were posted to all residents two weeks and six weeks after they received the survey.

#### Survey

The 91 item self-report survey was designed to include measures of the outcome variables (park usage, physical activity and active travel), potential determinants of these outcomes and relevant covariates. Respondents with a child(ren) aged 2–15 years living in the household, were asked to consider the child in the age range who had the next birthday and complete additional proxy-report survey questions regarding that child’s use of parks and related behaviours.

A socio-ecological framework [34] was used to develop the survey instrument which measured all three levels of influence on behaviours (intrapersonal, social and neighbourhood environmental influences). The survey included socio-demographic variables (age, sex, country of birth, marital status, employment status, highest level of education, motor vehicle access, dog ownership, time spent living at residential address and in the neighbourhood, and number of children) and participants self-reported their weight and height.

Where possible, established survey items from the literature with known reliability and validity were used. Self-reported transportation and leisure-time physical activity and time spent sitting in the last seven days was examined using the long form of the International physical activity questionnaire (IPAQ-L) [35]. Four items examined self-reported stress [36], ten items examined self-reported depression. CES-D10 [37] and a single item examined self-reported general health using the Short Form Health Survey (SF-36) (http://www.rand.org/health/surveys_tools/mos/mos_core_36item.html).

Nine existing items assessed perceptions of neighbourhood safety, violence, crime, attractiveness, opportunities to be active and walkability [38]: I feel safe walking in my neighbourhood, day or night; Violence is not a problem in my neighbourhood; My neighbourhood is safe from crime; My local neighbourhood is attractive; My neighbourhood offers many opportunities to be physically active; Local sports clubs and other facilities in my neighbourhood offer many opportunities to get exercise; Strange danger is a concern of mine; It is pleasant to walk in my neighbourhood; and, In my neighbourhood it is easy to walk to places. Two items examined social norms: I often see other people walking in my neighbourhood; and, I often see other people exercising in my neighbourhood [38]. Five items examined social trust and cohesion: People in this neighbourhood can be trusted; This is a close knit neighbourhood; People around here are willing to help their neighbours; People in this neighbourhood generally don’t get along with each other; and, People in this neighbourhood do not share the same values [39]. Additional items were based on concepts of social networks: I know many people in this neighbourhood; My child has many friends in this neighbourhood; and, There are not many other children around for my child to play with. Sense of community was also assessed with two items: This neighbourhood is a good place to live; and, This neighbourhood is a good place to raise children [40].

Items relating to adult’s park use, perceptions of parks, importance of park features for physical activity, and activity levels within parks are outlined in detail in Additional file 1: Table S1. Parent proxy-reported items on child’s park use, park satisfaction for children, child’s safety and independent mobility within the neighbourhood, and child’s time outdoors, physical activity and sedentary activities are detailed in Additional file 2: Table S2. Test-retest reliability of these items are also presented in these Tables. To examine test-retest reliability, 200 reliability surveys were mailed to residents in the Westerfolds area who had already returned a completed survey; 126 reliability surveys were returned (63% response rate). The reliability of the items were examined using one-way single measure intra-class correlation coefficient for continuous variables (ICC) and percent agreement for categorical variables. An ICC of ≥0.75 was considered excellent and 0.4-0.74 was considered good [41]. Percent agreement values were also calculated and were considered acceptable if above 66% [42]. Almost all items (95%, 42 of 44) had at least acceptable percent agreement and 93% of items (64 of 69) demonstrated good test-retest reliability (ICC ≥ 0.40). Overall, the test re-test reliability for the park-related items were acceptable for use at each of the time-points and for future studies.

### Translation

As a large proportion (14.2%) of residents within the City of Brimbank speak Vietnamese at home, the survey was also translated into Vietnamese. The survey was distributed to residents and school families surrounding Brimbank Park in English, accompanied by a letter written in Vietnamese stating that a copy of the survey in Vietnamese could be requested. Open-ended responses written in Vietnamese were translated in-house by a Vietnamese speaking research assistant.

#### Sample size calculations

Sample size calculations for the neighbourhood survey were based on ordered logistic regression analyses from a previous pilot natural experiment study in parks [32] by comparing responses for use of control and intervention parks post-refurbishment. This analysis yielded an estimated Odds Ratio (OR) across seven categories of frequency of park use (i.e. from ‘most days’ to ‘have not visited in the past 6 months’) of 4.74 [2.71, 8.33]. The proportional odds assumption was violated (p = .026), meaning that the OR at each category was not similar enough to summarise into one OR, therefore, generalised ordered logistic regression calculating six OR’s across seven categories was performed. Using a conservative approach, the smallest individual OR (1.41) was used in the following calculations. With alpha = 0.05 (two-tailed), beta = 0.20, this gives a required sample size of 444 at each time point [43] for both adults and children.

#### Baseline response rate

From the 9649 surveys distributed, 1488 surveys were returned completed, with 866 surveys including data on children. A total of 4637 surveys were distributed via pre-schools and schools with 713 returned (15.4% response rate). A total of 4984 surveys were distributed via the two Councils with 772 returned (15.5% response rate) and 22.5% including responses about children. Twenty-eight Vietnamese surveys were requested; however, only three (11% response rate) were returned completed.

#### Compensation

Each of the schools received a $75 book voucher as compensation for their time. All participants who returned a survey were included in a draw for one of two $500 gift vouchers.

### Direct observation of park users

To assess park usage and park-based physical activity, direct observations of park users was conducted at T1, and will be repeated at T2 and T3, using the SOPARC (the System for Observing Play and Recreation in Communities) [44]. This is a reliable instrument for assessing physical activity in community settings and recent studies have used SOPARC to specifically assess physical activity in parks [45,46]. This instrument is based on momentary time sampling and involves undertaking systematic observations (scans) of each participant within the park at a particular time.

At T1, at each park, six trained observers conducted observations of ten clearly defined target areas, which included the playground, walking/cycling paths, and open space areas. Target areas were pre-determined and comparable between the intervention and control parks and the same target areas will be targeted at T2 and T3. During each scan, observers recorded each individual within view in their target area according to: their broad age group (i.e. child (1-12 yrs), teen (13-20 yrs), adult (21-59 yrs), or older adult (60 yrs+); sex (male or female); and the activity they were engaged in (i.e. lying down, sitting; standing; moderate; or vigorous activity). During weekdays, observations were conducted every hour from 7:30 am to 4:30 pm (except for one day where observations concluded at 1:30 pm due to rain), and on weekend days every hour from 8:30 am to 4:30 pm. Data were collected for a total of eight days, including four weekdays and four weekend days. This equated to a total of 1460 scans across the two parks. Scheduling of daily observations was consistent between the intervention and control parks and observations were not conducted on days of forecasted rain.

### Park user intercept interviews

Face-to-face intercept interviews were completed at T1, and will be repeated at T2 and T3 with English-speaking adult park users on days when observations are conducted. The items included in this short (5–10 minute) interview are outlined in Table 1.

**Table 1:** Variables assessed in the intercept survey

**Socio-demographic variables**	**Response options**
Age	Years
Sex	Male/Female
Suburb of residence	Open ended
Time living at current address	Years
Do you own a dog?	Yes/No
Do you have a child <2 years?	Yes/No
Do you have a child 2–15 years?	Yes/No
**Park use**	
What is your main reason for visiting the park today?	1) walk, 2) walk the dog, 3) jog, 4) ride a bike, 5) ball games, 6) exercise, 7) supervise children, 8) take children to playground, 9) relax, 10) picnic, 11) socialise, 12) attend major event/celebration, 13) visit café, 14) view nature, 15) other
Why did you visit this park instead of other parks?	Open ended
How long do you plan to stay in the park today?	1) <30 mins, 2) 30 mins-1 hr, 3) 1 < 2 hrs, 4) 2 < 3 hrs, 5) 3 < 4 hrs, 6) 4+ hrs
Who came with you to the park today?	1) alone, 2) partner, 3) child(ren), 4) grandchildren, 5) friends, 6) organised group, 7) dog, 8) other
How did you get to this park today?	1) walked, 2) cycled, 3) public transport, 4) car, 5) jogged, 6) other
Did you come to the park from your home?	Yes/No
How long did it take for you to get to this park today?	Minutes
In the past 3 months, on average, how often have you visited this park?	1) daily, 2) 2–3 times per week, 3) once per week, 4) 2–3 times per month, 5) once per month, 6) < once per month, 7) first time to this park
In the past 3 months, which describes your usual activity levels during your visits to this park?	1) mostly sitting, 2) mostly light activities, 3) mostly moderate activities, 4) mostly vigorous activities
What could be done to encourage you to visit this park more often?	Open ended
**Satisfaction with the park**	
I am satisfied with:	
The quality of the park	1) strongly disagree, 2) disagree, 3) neither agree or disagree, 4) agree, 5) strongly agree 6) don’t know
The facilities available	As above
The playground	As above
The walking/cycling tracks	As above
The maintenance of the grounds and facilities	As above
The dog walking facilities	As above
**Health-related measures**	
In general, would you say your health is	1) excellent, 2) very good, 3) good, 4) fair, 5) poor
Child variables*	
Age	Years
Sex	Male/Female
Are they with you in the park today?	Yes/No
Have they ever visited this park?	Yes/No
In the past 3 months, on average, how often have they visited this park?	1) daily, 2) 2–3 times per week, 3) once per week, 4) 2–3 times per month, 5) once per month, 6) < once per month, 7) first time to this park
What do they usually do when at this park?	Open ended
Who do they usually come with to this park?	1) alone, 2) adult, 3) friends, 4) organised group, 5) dog, 6) other

*Proxy-reported by adult for child aged 2–15 years who has the next birthday.

At T1, trained, clearly identifiable research assistants approached park users in the specified target areas, explained the study and all ethical considerations, and invited participation. Participants were also asked if they had a child(ren) aged 2–15 years, and if so, they were asked to consider the child in the age range who had the next birthday and answer additional questions relating to this child’s use of the park. Overall, 794 park users agreed to participate (75.3% of those approached, excluding those who had already completed an interview). A total of 313 interviews were conducted at the intervention park (73.6% of those approached) and 481 interviews at the control park (76.5% of those approached). The child questions were completed by 366 (46.1%) participants overall. The main reasons for non-participation apart from already having completed the survey (44%) included: too busy/no time (21%), did not want to stop exercising (8%), were not interested (6%) and/or did not speak English (4%).

### Electronic path monitors and car traffic counters

Electronic path monitors were used at T1, and will be repeated at T2 and T3 to record the number of people walking and cycling on two pre-selected paths at both parks. The monitors incorporate the use of an infra-red beam and counter and are positioned at a height to include counts of children but to exclude counts of dogs (very large dogs may have been counted). At T1, two path monitors were positioned at the same location over the eight days of data collection at both parks. The path monitor location was decided by Parks Victoria rangers at each park, along with research staff. The locations were areas that were used most frequently and were most comparable between the two parks. Both parks incorporate a path that enables transport to the Melbourne CBD and one monitor in each park was positioned to record this traffic. The path monitors were set up at 7:30 am on weekdays and 8:30 am on the weekend days and were removed at 4:30 pm.

At T1 a traffic counter was located on the road at the entrance to both parks to record the number of vehicles entering and exiting the parks (hourly counts) over the 14-day period that incorporated the days when park observations were conducted. This will be repeated at T2 and T3.

## Discussion

The refurbishment of this large suburban park in a low SES area affords a unique opportunity to evaluate a ‘real world’ natural experiment. There is increasing interest in using the opportunities presented by natural experiments to generate evidence about population health impacts [47]. This study provides novel evidence for policy- and decision-makers regarding the role of parks for increasing community physical activity levels. Moreover, the location of the intervention park in a disadvantaged area will help inform the extent to which interventions of this type can beneficially influence the physical activity of those at higher risk in the population. Strengths of the study design include the wide range of comprehensive measures and the inclusion of partners which was integral to the data collection and will greatly assist the dissemination of results to key stakeholders. Previous research has suggested that building meaningful partnerships with diverse communities can improve health outcomes [48].

However, many challenges must be overcome in the development and implementation of this type of intervention study. One challenge of natural experiment research is timing the completion of measures with an unpredictable and frequently changing intervention timetable which is completely outside the researchers’ control [26]. The refurbishment works at Brimbank Park were delayed on numerous occasions, and significant negotiation and flexibility was required to co-ordinate the research funding availability with the intervention schedule. Typically, once grant money is awarded, data collection cannot be delayed indefinitely to accommodate delays in building works. Flexibility is therefore required by funding bodies to accommodate events outside of the researchers’ control.

An additional challenge is the ability to identify equally matched intervention and control parks. In the current study, it was not possible to match the parks according to area-level SES, hence the control park is located in a high SES neighbourhood and intervention park in a low SES area. However, given that the objective was to compare differences in changes in park use and in park-based physical activity between the intervention and control park, the study design will overcome this mismatch for area-level SES.

An additional uncontrollable factor was the constraint of weather. Although at T1 observation days with forecast rain were rescheduled (which required flexibility within the field team), weather conditions that were not severe enough to postpone data collection, such as light drizzle, very cold mornings and windy days, may have affected park usage. Although the unpredictable weather was a limiting factor, the forecast and actual weather conditions for each observation day were recorded and will be used when interpreting the results. Further, despite regular communication with park rangers, events often occurred in the park without our prior knowledge (i.e. burning areas of overgrowth for fire control, school excursions and community events) and such events may have resulted in atypical numbers of park-users. In addition, the large size of the intervention park meant that observers were unable to observe all park areas and observations were only conducted in the specified target to areas; however, the use of electronic monitors to record path usage and traffic will assist with monitoring of usage of the park outside the targeted observation areas.

## Conclusions

Despite these challenges, REVAMP will provide valuable unique insights into the impact of park refurbishment on park use and park-based physical activity and the development of natural experiment methodology. The findings will also provide information for future park developments and refurbishments and have the potential to assist urban planners and park designers to develop parks that are relevant to the communities they serve, attract users and encourage park users to be physically active.

## Competing interests

The authors declare that they have no competing interests.

## Authors’ contributions

JV conceived the study. JS, DC, AC, BGC and AT contributed to the study design and helped draft the manuscript. EF assisted with the data collection, data entry and helped draft the manuscript. All authors read and approved the final manuscript.

## Pre-publication history

The pre-publication history for this paper can be accessed here:

http://www.biomedcentral.com/1471-2458/14/600/prepub

## Supplementary Material

Additional file 1: Table S1 Items in the neighbourhood survey examining park use, and perceptions of parks among adults [49-52]. Click here for file

Additional file 2: Table S2 Parent proxy-reported items in the neighbourhood survey examining child’s use of parks, perceptions of parks, child’s safety and independent mobility in the neighbourhood, and child’s physical activity and sedentary activities [49-57]. Click here for file
