# Supplementary material for: A natural experiment to examine the impact of park renewal on park-use and park-based physical activity in a disadvantaged neighbourhood: the REVAMP study methods
Source: BMC Public Health. 2014 Jun 13;14:600. doi: 10.1186/1471-2458-14-600 (PMC4073813; doi:10.1186/1471-2458-14-600)
Supplement: Additional file 1: Table S1 — Items in the neighbourhood survey examining park use, and perceptions of parks among adults [49-52]. [file 1471-2458-14-600-S1.docx]

**Table 1: Items in the neighbourhood survey examining park use, and perceptions of parks among adults**

| **Item** | | **Response option** | **Reliability** | |
| --- | --- | --- | --- | --- |
|  |  |  | **% Agreement** | **ICC** |
| **Park use (intervention/control park)** | | | | |
| 1 | Have you heard of the intervention/control park? | Yes/No | 96.67 |  |
| 2 | Have you ever visited the intervention/control park? | Yes/No | 97.30 |  |
| 3 | In the past 3 months, on average, how often have you visited the intervention/control park? [[49](#_ENREF_49)]* | 1) daily, 2) 2-3 times/week,  3) once/week, 4) 2-3 times/month,  5) once/month, 6) <once/month,  7) have not visited in past 3 months |  | 0.85 |
| 4a | In the past 3 months, how long did you usually spend at the intervention/control park on weekdays? [[49](#_ENREF_49)]* | 1) <30mins, 2) 30-59mins, 3) 1<2hrs,  4) 2<3hrs, 5) 3<4hrs, 6) 4+ hrs, 7) N/A |  | 0.84 |
| 4b | In the past 3 months, how long did you usually spend at the intervention/control park on weekend days? [[49](#_ENREF_49)]* | As above |  | 0.85 |
| 5 | In the past 3 months, who did you usually go with when visiting the intervention/control park? [[49](#_ENREF_49)]* |  |  |  |
| 5a | Alone | Yes/No | 93.85 |  |
| 5b | Partner or adult family members | As above | 64.62 |  |
| 5c | Child(ren) | As above | 95.38 |  |
| 5d | Grandchild(ren) | As above | 96.92 |  |
| 5e | Friends | As above | 84.62 |  |
| 5f | Organised group | As above | 96.92 |  |
| 5g | Dog | As above | 87.69 |  |
| 6 | In the past 3 months, how did you usually get to the intervention/control park? | 1) walked, 2) cycled, 3) public transport, 4) car, 5) other | 92.31 |  |
| 7 | If you did not usually walk or cycle:  In the past 3 months, what is the main reason you did not usually walk or cycle to the intervention/control park? | 1) concern about traffic danger, 2) more convenient to travel by car or public transport, 3) too far away to walk or cycle there, 4) other | 65.0 |  |
| 8 | In the past 3 months, what activities did you usually do during your visits to the intervention/control park? [[49](#_ENREF_49)]* |  |  |  |
| 8a | Went for a walk (excluding dog walking) | Yes/No | 68.75 |  |
| 8b | Walked the dog | As above | 92.19 |  |
| 8c | Went for a jog/run | As above | 96.88 |  |
| 8d | Rode a bike | As above | 93.75 |  |
| 8e | Played ball games | As above | 90.63 |  |
| 8f | Did other exercise | As above | 98.44 |  |
| 8g | Supervised children | As above | 84.38 |  |
| 8h | Took children to playground | As above | 89.06 |  |
| 8i | Relaxed | As above | 73.44 |  |
| 8j | Had a picnic/BBQ | As above | 87.50 |  |
| 8k | Socialised with family/friends | As above | 76.56 |  |
| 8l | Attended a major event/celebration/birthday | As above | 98.44 |  |
| 8m | Visited a café/restaurant | As above | 92.19 |  |
| 8n | Viewed nature | As above | 79.69 |  |
| 9 | In the past 3 months, how long were you usually physically active for on each visit to the intervention/control park? [[49](#_ENREF_49), [50](#_ENREF_50)]* | Minutes |  | 0.84 |
| 10 | In the past 3 months, which of the following best describes your usual activity level during your visits to the intervention/control park? [[49](#_ENREF_49), [50](#_ENREF_50)]* | 1) mostly sitting, 2) mostly light activities, 3) mostly moderate activities, 4) mostly vigorous activities |  | 0.79 |
| **Perceptions of intervention/control park** | | | | |
| 11 | Six items examined satisfaction with intervention/control park:  How much do you agree/disagree with the following statements? |  |  |  |
| 11a | I am satisfied with the overall quality | 1) strongly disagree, 2) disagree,  3) neither agree or disagree, 4) agree,  5) strongly agree, 6) don’t know |  | 0.43 |
| 11b | I am satisfied with the facilities available | As above |  | 0.43 |
| 11c | I am satisfied with the playground | As above |  | 0.33 |
| 11d | I am satisfied with the walking/cycling tracks | As above |  | 0.49 |
| 11e | I am satisfied with the maintenance of the grounds and facilities | As above |  | 0.54 |
| 11f | I am satisfied with the dog walking facilities | As above |  | 0.42 |
| 12 | Four items examined perceptions of shade:  Considering trees, and covered/sheltered areas, how adequate is the shade provided for sun protection during activities at the following facilities at the intervention/control park? [[51](#_ENREF_51)]* |  |  |  |
| 12a | Picnic facilities | 1) no adequate shade, 2) shade available in some areas, 3) shade available in most areas, 4) substantial shade in all areas,  5) can’t say, 6) N/A |  | 0.37 |
| 12b | Seated areas | As above |  | 0.66 |
| 12c | Café outdoor areas | As above |  | 0.41 |
| 12d | Playground | As above |  | 0.62 |
| **Park use (other than intervention/control park)** | | | | |
| 13 | In the past 3 months, on average, how often have you visited a park (not including intervention/control park)? [[49](#_ENREF_49)]* | 1) daily, 2) 2-3 times/week,  3) once/week, 4) 2-3 times/month,  5) once/month, 6) <once/month,  7) have not visited in past 3 months |  | 0.79 |
| 14 | Have you visited a park in the past 7 days? | Yes/No | N/A |  |
| 15 | Items 3,4,5,8, 9,10 listed above were repeated for park visited most often in the past 3 months (not including intervention/control park) |  | N/A |  |
| **Perceptions of neighbourhood parks** | | | | |
| 16 | 12 items assessed perceptions of neighbourhood parks:  How much do you agree or disagree with the following statements? [[50](#_ENREF_50)]* |  |  |  |
| 16a | I am satisfied with overall quality of parks in my neighbourhood | 1) strongly disagree, 2) disagree,  3) neither agree or disagree, 4) agree,  5) strongly agree, 6) don’t know, 7) N/A |  | 0.47 |
| 16b | Parks in my neighbourhood are used by many people | As above |  | 0.46 |
| 16c | Parks in my neighbourhood are attractive | As above |  | 0.52 |
| 16d | Parks in my neighbourhood are safe | As above |  | 0.55 |
| 16e | Parks in my neighbourhood are well maintained | As above |  | 0.65 |
| 16f | Parks in my neighbourhood have satisfactory shade | As above |  | 0.62 |
| 16g | Parks in my neighbourhood have suitable dog walking facilities | As above |  | 0.49 |
| 16h | Parks in my neighbourhood have facilities I am interested in | As above |  | 0.36 |
| 16i | I have to cross a busy road to get to my closest park | As above |  | 0.72 |
| 16j | I am concerned about the presence of gangs or ‘hoons’ in parks | As above |  | 0.60 |
| 16k | I am concerned about the amount of graffiti/vandalism in parks | As above |  | 0.68 |
| 16l | My children like going to parks in my neighbourhood | As above |  | 0.60 |
| 17 | Three items examined time taken to walk to various parks:  About how long would it take to walk from home to the following places? [[52](#_ENREF_52)]* |  |  |  |
| 17a | Nearest park to home | 1) 1-5 mins, 2) 6-10 mins,  3) 11-20 mins, 4) 21-30 mins,  5) 31+ mins 6) don’t know |  | 0.72 |
| 17b | Park you visited most often in the past 3 months (not including intervention/control park) | As above |  | 0.80 |
| 17c | Intervention/control park | As above |  | 0.72 |
| 18 | 20 items assessed the importance of particular park features for encouraging park-based physical activity:  If you were going to do regular physical activity at a park in the next two weeks, how important would each of the following features be? |  |  |  |
| 18a | It is within walking distance from where you live or work | 1) not at all important, 2) not very important, 3) neither, 4) quite important, 5) very important |  | 0.49 |
| 18b | It is easy to get to | As above |  | 0.36 |
| 18c | It is close to public transport | As above |  | 0.54 |
| 18d | It has car parking | As above |  | 0.52 |
| 18e | It is used by people who are friendly | As above |  | 0.64 |
| 18f | You personally feel safe going there whenever you want to | As above |  | 0.43 |
| 18g | You often see other people using it | As above |  | 0.45 |
| 18h | It has attractive trees and birdlife | As above |  | 0.58 |
| 18i | It has a relaxing atmosphere | As above |  | 0.54 |
| 18j | It is well maintained | As above |  | 0.47 |
| 18k | It has an attractive feature like a lake or view | As above |  | 0.63 |
| 18l | There are interesting walks, cycles or jogs to do | As above |  | 0.41 |
| 18m | There are a variety of paths to take | As above |  | 0.45 |
| 18n | There are benches | As above |  | 0.53 |
| 18o | There are drinking fountains | As above |  | 0.65 |
| 18p | There are toilets | As above |  | 0.72 |
| 18q | Children’s play equipment is available | As above |  | 0.53 |
| 18r | There are shade trees | As above |  | 0.69 |
| 18s | There are bike racks | As above |  | 0.54 |
| 18t | There is a dog off-leash area | As above |  | 0.69 |

*Item modified from source; ICC=Intra class correlations
