# Supplementary material for: A natural experiment to examine the impact of park renewal on park-use and park-based physical activity in a disadvantaged neighbourhood: the REVAMP study methods
Source: BMC Public Health. 2014 Jun 13;14:600. doi: 10.1186/1471-2458-14-600 (PMC4073813; doi:10.1186/1471-2458-14-600)
Supplement: Additional file 2: Table S2 — Parent proxy-reported items in the neighbourhood survey examining child’s use of parks, perceptions of parks, child’s safety and independent mobility in the neighbourhood, and child’s physical activity and sedentary activities [49-57]. [file 1471-2458-14-600-S2.docx]

**Table 2: Parent proxy-reported items in the neighbourhood survey examining child’s use of parks, perceptions of parks, child’s safety and independent mobility in the neighbourhood, and child’s physical activity and sedentary activities**

| **Item** | | **Response option** | **Reliability** | |
| --- | --- | --- | --- | --- |
|  |  |  | **% Agreement** | **ICC** |
| **Child’s park use (intervention/control park)** | | | | |
| 1 | In the past 3 months, on average, how often has your child visited the intervention/control park? [[49](#_ENREF_49)]* | 1) daily, 2) 2-3 times/week,  3) once/week, 4) 2-3 times/month,  5) once/month, 6) <once/month,  7) have not visited in past 3 months |  | 0.73 |
| 2a | In the past 3 months, how long did your child usually spend at the intervention/control park on weekdays? [[49](#_ENREF_49)]* | 1) <30mins, 2) 30-59mins, 3) 1<2hrs,  4) 2<3hrs, 5) 3-4hrs, 6) 4+ hrs, 7) N/A |  | 088 |
| 2b | In the past 3 months, how long did your child usually spend at the intervention/control park on weekend days? [[49](#_ENREF_49)]* | As above |  | 0.92 |
| 3 | In the past 3 months, who did your child usually go with when visiting the intervention/control park? [[49](#_ENREF_49)]* |  |  |  |
| 3a | Alone | Yes/No | too few rating categories |  |
| 3b | Self or other adult family members | As above | too few rating categories |  |
| 3c | Siblings | As above | 75.0 |  |
| 3d | Friends | As above | 95.83 |  |
| 3e | Organised group | As above | too few rating categories |  |
| 3f | Dog | As above | 91.67 |  |
| 4 | In the past 3 months, how did your child usually get to the intervention/control park? | 1) pushed in pram or stroller, 2) walked, 3) cycled, 4) public transport, 5) car, 6) other | 95.83 |  |
| 5 | In the past 3 months, what activities did your child usually do when visiting the intervention/control park? [[49](#_ENREF_49)]* |  |  |  |
| 5a | Went for a walk | Yes/No | 75.00 |  |
| 5b | Walked the dog | As above | 100.00 |  |
| 5c | Went for a jog/run | As above | 83.33 |  |
| 5d | Rode a bike | As above | 66.67 |  |
| 5e | Played ball games | As above | 83.33 |  |
| 5f | Did other exercise | As above | 87.50 |  |
| 5g | Played on the playground | As above | 91.67 |  |
| 5h | Relaxed | As above | 83.33 |  |
| 5i | Had a picnic/BBQ | As above | 100.00 |  |
| 5j | Socialised with family/friends | As above | 66.67 |  |
| 5k | Attended a major event/celebration/birthday | As above | 95.83 |  |
| 5l | Visited cafe | As above | 91.67 |  |
| 5m | Viewed nature | As above | 75.00 |  |
| 6 | In the past 3 months, how long was your child usually physically active for on each visit to intervention/control park? [[49](#_ENREF_49), [50](#_ENREF_50)] | Minutes |  | 0.43 |
| 7 | In the past 3 months, which of the following best describes your child’s usual activity level during visits to intervention/control park? [[49](#_ENREF_49), [50](#_ENREF_50)]* | 1) mostly sitting, 2) mostly light activities, 3) mostly moderate activities, 4) mostly vigorous activities | 89.58 |  |
| **Child’s park use (other than intervention/control park)** | | | | |
| 8 | In the past 3 months, on average, how often has your child visited a park (not including intervention/control park)? | 1) daily, 2) 2-3 times/week, 3) once/week,  4) 2-3 times/month, 5) once/month,  6) <once/month,  7) have not visited in past 3 months |  | 0.81 |
| 9 | Has your child visited a park in the past 7 days? | Yes/No | N/A |  |
| 10 | Items 1,2,3,5,6,7 listed above repeated for park visited most often in the past 3 months (not including intervention/control park) | N/A | N/A |  |
| **Perceptions of intervention/control park and neighbourhood parks** | | | | |
| 11 | How much do you agree with the following statements? |  |  |  |
| 11a | I am satisfied with the quality of intervention/control park for my child | 1) strongly disagree, 2) disagree,  3) neither agree or disagree, 4) agree,  5) strongly agree, 6) don’t know |  | 0.54 |
| 11b | I am satisfied with the quality of the play equipment at the intervention/control park for my child | As above |  | 0.54 |
| 11c | I am satisfied with the overall quality of available parks for my child in my neighbourhood | As above |  | 0.53 |
| 11d | I am satisfied with the overall quality of play equipment available for my child in my neighbourhood | As above |  | 0.53 |
| 12 | Are there parks/playgrounds that are within walking distance from home for your child? | Yes/No | 94.40 |  |
| **Child’s neighbourhood safety** | | | | |
| 13 | Four items examined neighbourhood safety:  How much do you agree with the following statements about your local neighbourhood? |  |  |  |
| 13a | My neighbourhood is safe for my child | 1) strongly disagree, 2) disagree,  3) neither agree or disagree, 4) agree,  5) strongly agree, 6) don’t know |  | 0.50 |
| 13b | My neighbourhood is safe for my child to walk/cycle around in the daytime | As above |  | 0.35 |
| 13c | My child would be safe walking home from a bus or train stop at night | As above |  | 0.60 |
| 13d | It is safe for my child to play or hang out in the street outside our house | As above |  | 0.56 |
| **Child’s independent mobility** | | | | |
| 14 | How often does your child usually walk to nearby parks/playgrounds with the following people? [[53](#_ENREF_53)]* |  |  |  |
| 14a | By him/herself | 1) never, 2) rarely, 3) sometimes, 4) often, 5) very often |  | 0.78 |
| 14b | With parent/other adult | As above |  | 0.45 |
| 14c | With friends/siblings | As above |  | 0.69 |
| 15 | How far away from home is your child allowed to roam on their own? [[53](#_ENREF_53)]* | 1) > 15 min walk from home, 2) within a 15 min walk from home, 3) within 2-3 streets away from home, 4) within my street, 5) my child is not allowed out alone |  | 0.82 |
| 16 | How far away from home is your child allowed to roam with friends (unaccompanied by an adult)? [[53](#_ENREF_53)]* | As above |  | 0.70 |
| **Child’s physical activity and sedentary activities** | | | | |
| 17 | During the last 7 days, how many hours/minutes in total did your child spend outside (Monday to Friday)? [54-55 ] | Hours and minutes | Not calculated |  |
| 18 | During the last 7 days, how many hours/minutes in total did your child spend outside (Saturday and Sunday)?[54-55] | As above | As above |  |
| 19 | Over the past 7 days, on how many days did your 5-15 year old child participate in sport, physical activity, or active play for a total of at least 60 minutes per day? [56 ]* | 1) 0 days, 2) 1 day, 2) 2 days, 3) 3 days, 4) 4 days, 5) 5 days, 6) 6 days, 7) 7 days | As above |  |
| 20 | Over the past 7 days, on how many days did your 5-15 year old watch television, play electronic games, or use the computer for less than 2 hours per day? [56]* | As above | As above |  |
| 21 | Over the past 7 days, on how many days did your 2-5 year old child (who has not yet started school) participate in active play for a total of at least 3 hours per day? | As above | As above |  |
| 22 | Over the past 7 days, on how many days did your 2-5 year old child (who has not yet started school) watch television, play electronic games, or use the computer for less than 1 hour per day? | As above | As above |  |
| 23 | Over the past 7 days, which of the following leisure activities did your child do? [57] |  | As above |  |
| 23a | Watch TV/DVDs | Total hours Monday-Friday and Saturday and Sunday |  |  |
| 23b | Played video games while seated | As above |  |  |
| 23c | Played video games while standing/active | As above |  |  |
| 23d | Used the computer/internet for educational purposes | As above |  |  |
| 23e | Used the computer/internet for non-educational purposes | As above |  |  |

*item modified from source; ICC=Intra class correlation
